# Simple Reconfigurable Circularly Polarized Antenna at Three Bands

**DOI:** 10.3390/s19102316

**Published:** 2019-05-20

**Authors:** Tu Tuan Le, Han-Young Park, Tae-Yeoul Yun

**Affiliations:** Department of Electrical and Computer Engineering, Hanyang University, Seoul 133-791, Korea; tuantu90@gmail.com (T.T.L.); wow1000wow@hanyang.ac.kr (H.-Y.P.)

**Keywords:** broadband, circularly polarized, parasitic element, reconfigurable antenna, triple-band

## Abstract

In this paper, a simple design for a triple-band circularly polarized (CP) antenna with the capability of switching its polarization between dual-sense CPs is presented. The proposed antenna is comprised of a monopole loop antenna as a primary radiator. By placing a parasitic loop around the primary radiator, an additional CP band is achieved. Reconfigurability of the polarization between right-hand CP (RHCP) and left-hand CP (LHCP) at three different frequencies of 2.5, 3.3, and 3.8 GHz was realized by controlling the ON/OFF states of two PIN diodes. For validation, the fabricated antenna yielded an impedance bandwidth of 52.6% (2.34–4.01 GHz), while the axial ratio bandwidths for both the RHCP and LHCP states were 3.5% (2.47–2.56 GHz), 6.6% (3.20–3.42 GHz), and 2.4% (3.74–3.83 GHz). The measured broadside gains within the axial ratio bandwidth were 1.2, 2.7, and 1.4 dBi, respectively. Compared to other reconfigurable multi-band CP antennas, the proposed design is the first work to achieve a reconfigurable polarization at three distinct bands at a low fabrication cost by using only two diodes. The proposed antenna is suitable for WLAN and WiMAX applications.

## 1. Introduction

Circularly polarized antennas with outstanding features exhibit many advantages over linearly polarized (LP) antennas, including the ability to mitigate polarization mismatch and establish stable communication between the transmitter and receiver sides. In recent years, circularly polarized (CP) antennas with the capability of switching polarization between right-hand CP (RHCP) and left-hand CP (LHCP) states have increasingly been used in wireless communication systems. In addition, this antenna type is also desirable for single antennas that can operate with multiple wireless applications. 

To achieve these needs, the first solution is to design antennas with reconfigurable polarization and wideband characteristics [1,2,3,4,5,6,7,8]. Array antennas adopted a Wilkinson power divider [1] and a power divider with a phase shifter [2] to obtain a wide axial-ratio bandwidth (ARBW). These antennas, however, require complex feeding networks. Other antennas with simple feeding methods were explored in [3,4,5,6,7,8]. These could consist of an electric dipole [3] or the combination of an electric dipole and a magnetic dipole [4,5]. These have a tapered configuration or a wide open-end configuration, which excites multimode resonances to achieve the wideband CP. Instead of using a PIN-diode as an electrical switching technique [1,2,3,4,5], other interesting techniques were proposed in [6,7]. The water spiral structure was used for the wideband CP performance in [6]. A glass container with two inversed channels is used to support the water spiral. By controlling the water flow among each of the two channels, the polarization of the antenna is tuned. In addition, microelectromechanical system (MEMS) switches on an E-shaped patch radiator were utilized to achieve the CP reconfigurability [7]. The two gaps in the E-shaped patch were used to create different electrical paths, which excited multimode resonances to achieve a wide band characteristic. Compared to the PIN diode, the water spiral structure and MEMS switches seem to cause difficulty in the fabrication process and/or increase the system cost. In addition, it has been known that one of the notable drawbacks of wideband antennas is interference with other radio communication systems. 

To tackle this problem, multi-band reconfigurable antennas were investigated. In addition, the features of a bi-directional radiation pattern and a low profile are required for some applications, in which monopole or slot structures were commonly utilized [8,9,10]. Multi-band reconfigurable CP antennas using the monopole structure were published in [11,12]. By controlling the state of four PIN diodes placed between the feedline and four radiating arms, the sense of polarization was interchanged at two CP bands [11]. Alternatively, by reconfiguring the location and direction of the L-shaped slot, the CP state was controlled using two PIN diodes at two CP bands [12]. The number of reconfigurable CP bands of both antennas were limited to two, and/or many switching diodes were used.

In this research, a simple yet effective method to realize a multi-band CP antenna with reconfigurable polarization and bi-directional radiation characteristics is presented. The proposed design is able to exhibit dual-sense CP realization at three different frequencies. By switching the state of two PIN diodes, the direction of currents flowing on the radiating element can be adjusted, and therefore, the sense of polarization can be changed. The proposed antenna is designed to operate at a single WLAN band (2.5 GHz) and partial WiMAX bands (3.3/3.8 GHz). The details of the CP excitation principle, antenna design procedure, and measured results are discussed in the following sections. 

## 2. Antenna Design

### 2.1. CP Excitation Principle

**Figure 1:**
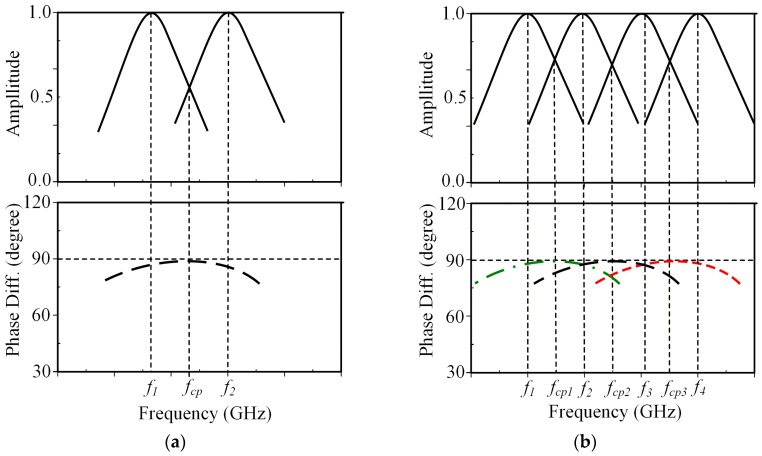
Characteristics of CP excitation: (**a**) single CP mode; (**b**) multi-CP modes.

The patch or ring antenna has generally been used for utilizing CP radiation due to its advantages of light weight, low fabrication cost, and versatility in shape. CP radiation can be obtained by introducing perturbation, which excites two linear polarization (LP) modes with a 90° phase difference. The easiest way to ideally excite CP is to feed the radiator at two adjacent edges by using a 90° power divider or 90° hybrid [13]. This technique, however, increases the complexity of the feeding method. A truncated corner or a diagonal slot is widely used for the square or circular patch antenna to achieve perturbation [14,15,16]. Meanwhile, a loaded gap was popularly used for the square-ring radiator [17,18,19,20]. These methods excite two adjacent orthogonal modes in the diagonal planes of equal amplitude and in quadrature phase [21] as shown in Figure 1a. The resonant frequency (*f_cp_*) of the CP mode can be roughly calculated using:

(1) $$f_{cp}\approx \frac{f_{1}+f_{2}}{2}$$

From this point of view, a potential method for achieving multi-CP bands is made by exciting multi-sets of two orthogonal LP modes as shown in Figure 1b. Similar to the case of single CP mode, the multi-CP resonant frequencies at *f*_*cp*1_, *f*_*cp*2_, *f*_*cp*3_ are approximated as the middle frequency of two adjacent LP modes as follows:

(2) $$f_{cp1}\approx \frac{f_{1}+f_{2}}{2}$$

(3) $$f_{cp2}\approx \frac{f_{2}+f_{3}}{2}$$

(4) $$f_{cp3}\approx \frac{f_{3}+f_{4}}{2}$$

### 2.2. Antenna Configuration and Design Procedure

**Figure 2:**
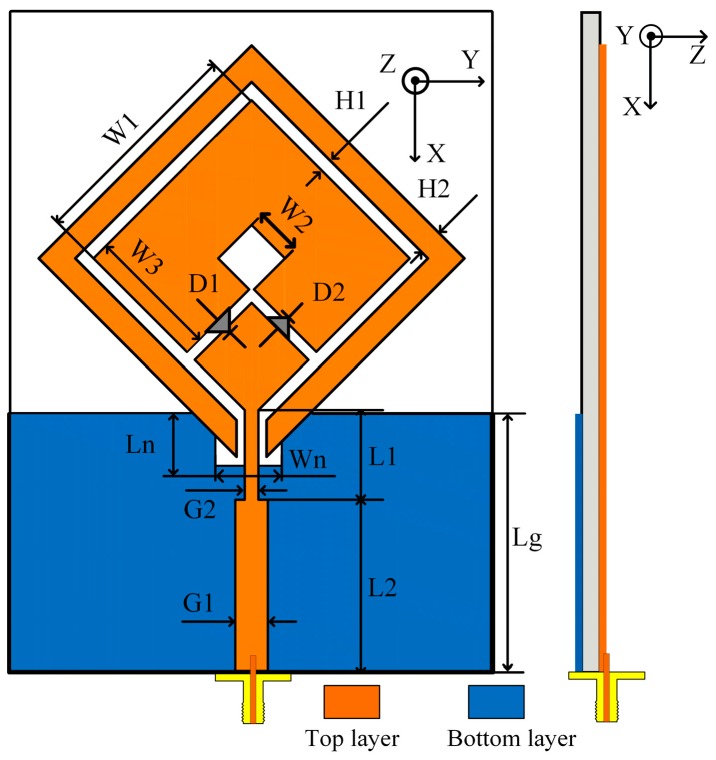
Geometry of the proposed antenna with *W*_1_ = 26, *W*_2_ = 5.5, *W*_3_ = 15.35, *L_g_* = 26, *H*_1_ = 1.5, *H*_2_ = 2.75, *L*_1_ = 10.4, *L*_2_ = 16.9, *G*_1_ = 3.8, *G*_2_ = 1.6, *W_n_* = 7, and *L_n_* = 1 (unit: mm).

**Figure 3:**
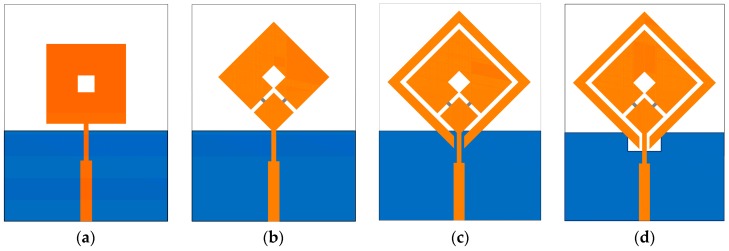
Evolutional procedure for the proposed antenna: (**a**) STEP 1; (**b**) STEP 2; (**c**) STEP 3; (**d**) STEP 4.

**Figure 4:**
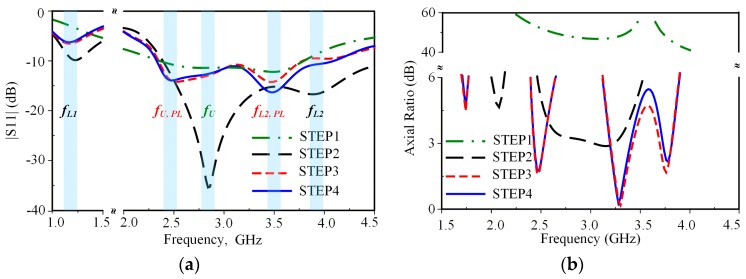
Improvement of antenna characteristics for four design steps: (**a**) S11; (**b**) axial ratio.

The geometry of the proposed antenna is illustrated in Figure 2. The proposed triple-band reconfigurable CP antenna is formed of a square-ring radiator, a parasitic loop, PIN diodes, and a notched ground plane. The proposed antenna was printed on a 70 × 55 × 1.57 mm^3^ Rogers RT/droid 5800 with a dielectric constant of 2.2 and a loss tangent of 0.0009. The evolution process and the simulated S11 and axial ratio (AR) of the proposed antenna for each step are shown in Figure 3 and Figure 4, respectively. The antenna was simulated using the commercial ANSOFT High Frequency Structure Simulator (HFSS). A detailed description of each step is listed as follows. 

STEP 1: A closed-loop square-ring radiator, denoted as an unloaded radiator, is fed by a 50-Ω transmission line. The closed-loop radiator generates an unloaded mode TM_11_ around 2.7 GHz (denoted as *f*_U_) as shown in Figure 4a with linearly polarized characteristics where AR > 40 dB, as shown in Figure 4b. For a conventional square-ring antenna, the resonances are estimated using the following equation [21]:

(5) $$f_{11}=\frac{nc}{4W_{1}\sqrt{\varepsilon_{r}}}\;n=1,\;2,\;3\ldots$$

where $\varepsilon_{r}$ is the effective dielectric constant, *c* is the velocity of light, and *W*_1_ is the outer length of the square-ring. 

STEP 2: In this step, the square-ring is rotated 45° around the *z* axis and fed at one corner. Two PIN diodes, *D*_1_ and *D*_2_, are inserted into the square-ring radiating arm as shown in Figure 3b. When DC voltage is applied, one PIN diode is in an ON state and the other is in an OFF state. The square-ring radiator loaded with a gap or PIN diodes is denoted as the loaded radiator. The loaded radiator additionally generates two loaded modes (denoted as *f_L_*_1_ and *f_L_*_2_), where *f_L_*_1_ < *f_U_* < *f_L_*_2_ [22] as shown in Figure 4a. Two loaded modes, *f_L_*_1_ and *f_L_*_2_, with an unloaded mode, *f_U_*, created two CP modes. As a result, the CP modes at 2.1 and 3.3 GHz were excited: 2.1 GHz ≈ (*f*_*L*1_ + *f_U_*)/2 and 3.3 GHz ≈ (*f_L_*_2_ + *f_U_*)/2. In addition, a CP mode occurred at a frequency around 2.5 GHz, which is excited by the intense surface currents near the inner edge of the loaded square-ring. 

STEP 3: A parasitic loop with the width dimension of *H*_2_ was added. The gap, *H*_1_*,* is used to adjust the electromagnetic coupling between the parasitic loop and square-ring. The parasitic loop additionally generates new unloaded (*f_U_, _PL_*) and two loaded modes (*f_L1, PL_*, *f_L_*_2_, *_PL_*) as shown in Figure 4a. The presence of the parasitic loop degrades S11 at *f_U_*, *f_L_*_1_, and *f_L_*_2_ of the loaded square-ring radiator. As a result, the CP mode at 2.1 GHz is significantly deteriorated where AR > 10 dB. Based on the study in [23], the stack method was presented for generating a CP mode by combining unloaded and loaded modes of two loaded square-rings utilizing two layers of substrate. In this research, however, dual-CP modes are excited by two new sets of two orthogonal LP modes. Evidently, the CP modes at 1.7 and 3.8 GHz are newly excited from two sets of two orthogonal LP modes (*f_L_*_1_ + *f_U_, _PL_*) and (*f_L__2_, _PL_* + *f_L_*_2_) as shown in Figure 4, respectively. However, only CP modes targeted for WLAN and WiMAX applications at 2.5, 3.3, and 3.8 GHz are below 3-dB AR, satisfying the CP radiation requirement. Herein, we should note that the −10-dB impedance bandwidth (IBW) of 45.0% (2.34–3.70 GHz) does not fully cover the 3-dB AR bandwidth (ARBW) of 4.0% (2.43–2.53 GHz), 6.4% (3.20–3.41 GHz), and 3.2% (3.69–3.81 GHz). 

STEP 4: To further improve the IBW, STEP 4 is performed. A technique of using notch ground plane to fine-detune the antenna’s input impedance, which has been thoroughly investigated in [24], is applied. The notch ground only affects IBW at a high frequency region, while ARBW is almost unaffected, as shown in Figure 4. The best antenna performance was achieved with notch dimensions *W_n_ × L_n_* of 7 × 0.7 mm^2^. As a result, the proposed antenna yields a wide IBW of 55.2% (2.32–4.09 GHz), which fully covered the ARBW. 

Table 1 summarizes the generation of unloaded, loaded, and CP modes from STEP 1 to STEP 4. Noted that while the proposed antenna excites four distinct CP modes, only the ARBWs of three modes—at frequencies of 2.5, 3.3, and 3.8 GHz—are below 3-dB. 

**Figure 5:**
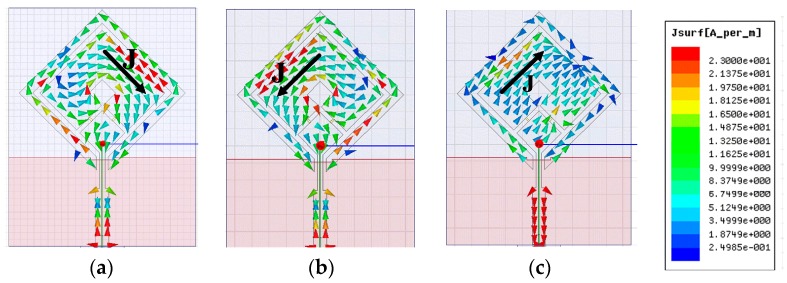

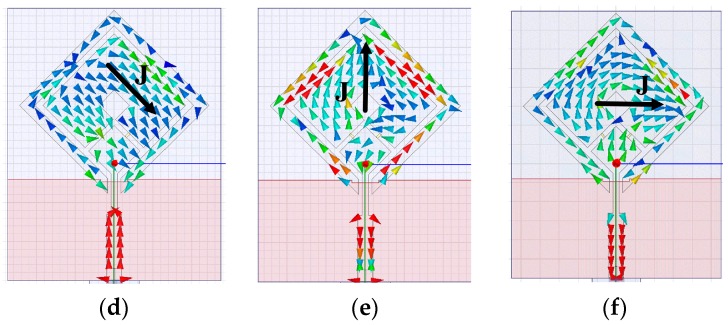
Current distributions to demonstrate the LHCP operation at three frequencies (*D*_1_ ON, *D*_2_ OFF): (**a**) 2.5 GHz, t = 0; (**b**) 2.5 GHz, t = T/4; (**c**) 3.3 GHz, t = 0; (**d**) 3.3 GHz, t = T/4; (**e**) 3.8 GHz, t = 0; (**f**) 3.8 GHz, t = T/4.

To enable us to further understand the antenna’s CP operation mechanism, the current distributions on the antenna at three frequencies of 2.5, 3.3, and 3.8 GHz are shown in Figure 5. The results were simulated at two different times of t = 0 and t = T/4, where T is the period and J is the vector summation of the major current components. As observed at a frequency of 2.5 GHz, vector J at both t = 0 and t = T/4 is orthogonal in direction and equal in magnitude, as shown in Figure 5a,b. Similar observations were made for 3.3 and 3.8 GHz as shown in Figure 5c–f, respectively. At three frequencies, vector J rotates clockwise with time as observed from the +*z* direction. As a result, the proposed antenna radiates LHCP radiation when *D*_1_ ON and *D*_2_ OFF.

### 2.3. Parametric Study

This section investigates the effect of key parameters on antenna performance. The outer (*W*_1_) and inner (*W*_2_) lengths of the square-ring and the parasitic loop width (*H*_2_), which determine the circumference of the square-ring and parasitic loops, are considered. During variation of a parameter, the other parameters are fixed at their optimized values, as mentioned in the caption of Figure 2. However, varying *W*_1_ changes both the square-ring and parasitic loop circumferences. In order to more clearly observe the influence of circumference, the parameter of *H*_1_ is adjusted while *W*_1_ is varied.

**Figure 6:**
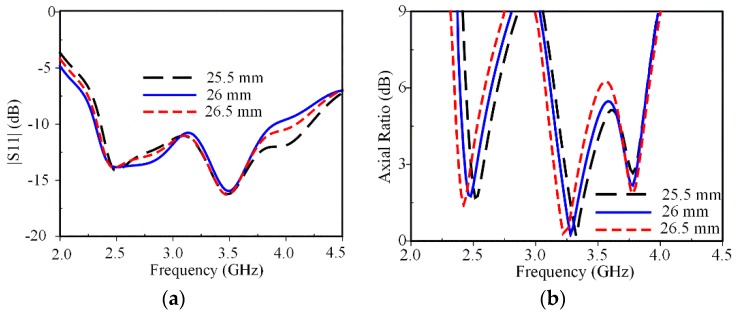
Effect of *W*_1_ on antenna performances: (**a**) S11; (**b**) axial ratio.

Figure 6 shows the effect of *W*_1_ on antenna performance. Varying *W*_1_ affects the unloaded mode *f_U_* (2.8 GHz) and loaded mode *f_L_*_2_ (4.0 GHz), which has a strong effect on the CP modes at 2.5 and 3.3 GHz and a negligible effect on the CP mode at 3.8 GHz. Increasing *W*_1_ not only increases the circumference of the loaded square-ring but also increases the electrical path of the surface currents distributed on the parasitic loop due to the electromagnetic coupling, thus shifting the CP modes at 2.5 and 3.3 GHz to a lower frequency. The optimal ARBW is obtained for the value of *W*_1_ equal to 26 mm. 

**Figure 7:**
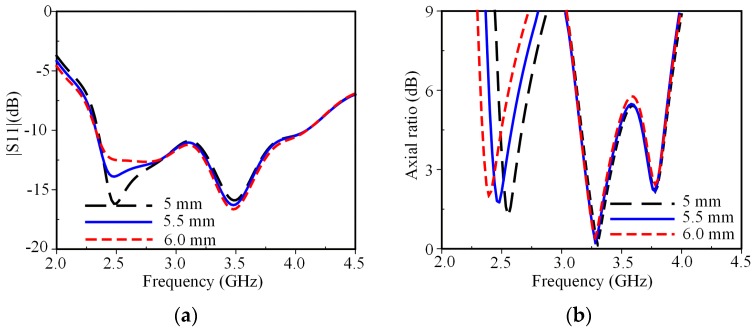
Effect of *W*_2_ on antenna performances: (**a**) S11; (**b**) axial ratio.

Figure 7 shows the antenna’s performance on *W*_2_ variation. S11 and AR of the CP mode at 2.5 GHz are sensitive to the variation of *W*_2_, while the antenna’s performance at middle and higher frequencies are independent of the variation of *W*_2_. As *W*_2_ increases, the circumference of the inner loop increases, and thus, the CP mode at 2.5 GHz moves to a lower frequency. This also confirms that the CP mode at 2.5 GHz is excited by the surface currents near the inner edge of the loaded square-ring. The optimal value of *W*_2_ is determined to be 5.5 mm. 

**Figure 8:**
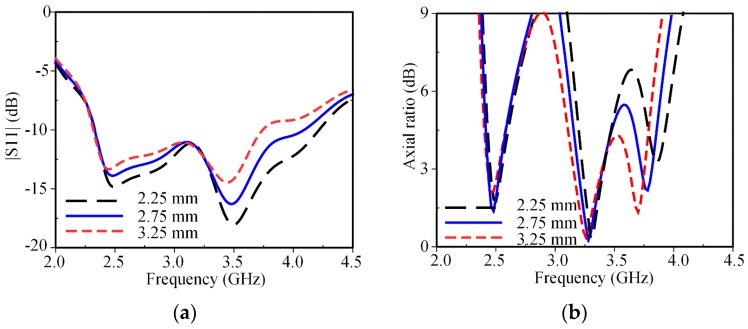
Effect of *H*_2_ on antenna performances: (**a**) S11; (**b**) axial ratio.

Figure 8 shows that the parasitic loop can be used to adjust the antenna’s input impedance, particularly at higher frequencies. The variation of *H*_2_ significantly affects the CP mode at 3.8 GHz, while it slightly affects the CP mode at 3.3 GHz and rarely affects the CP mode at 2.5 GHz. As *H*_2_ decreases, the outer edge circumference of the parasitic loop decreases, and thus, the AR mode at 3.8 GHz shifts to a higher frequency. In addition, as *H*_2_ varies, the antenna’s input impedance varies, and thus, the S11 curve is changed over the entire bandwidth. It is observed that the best CP performance along with the best IBW is achieved at *H*_2_ of 2.75 mm. 

As a result, the parametric study shows that three CP modes at 2.5, 3.3, and 3.8 GHz can be controlled by *W*_1_, *W*_2_, and *H*_2_.

### 2.4. PIN-Diode and Switching of the CP-Sense

The capability of switching between LHCP and RHCP at triple CP bands is realized by employing two PIN-diodes (*D*_1_, *D*_2_), Microsemi MPP4203 [25], which are used to route the current’s flow in different paths. Simplified equivalent circuits of the diodes were used for the simulation, which were obtained using the Thru-Reflect-Line (TRL) calibration: a resistor (*R_S_* = 3.5 Ω) and an inductor (*L_S_* = 0.45 nH) in series for the ON state, a capacitor (*C_T_* = 0.08 pF) and a resistor (*R_T_* = 3 kΩ) in parallel and series with an inductor (*L_T_* = 0.45 nH) for the OFF state. The PIN diodes were biased using a Bias-T from a 3-V battery source. The upper part of the square-ring was connected to the battery through a via and a strip line. A surface-mount inductor of 18 nH was adopted for the RF choke to isolate the RF signal from the DC circuit. Table 2 shows that CP reconfigurability is achieved at three bands by switching only two PIN-diodes.

## 3. Experiment Results

**Figure 9:**
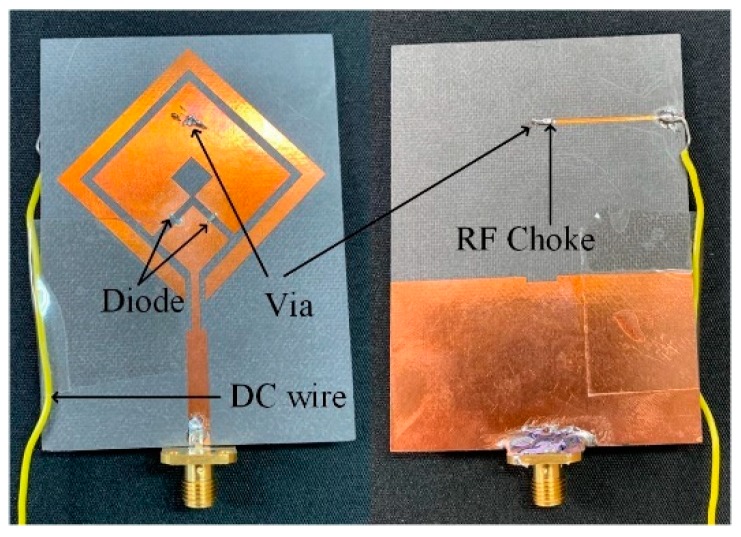
Photograph of fabricated antenna.

The triple-band reconfigurable circularly polarized antenna was fabricated and verified by measurement in an anechoic chamber. The fabricated antenna and details of biasing arrangement are shown in Figure 9. 

**Figure 10:**
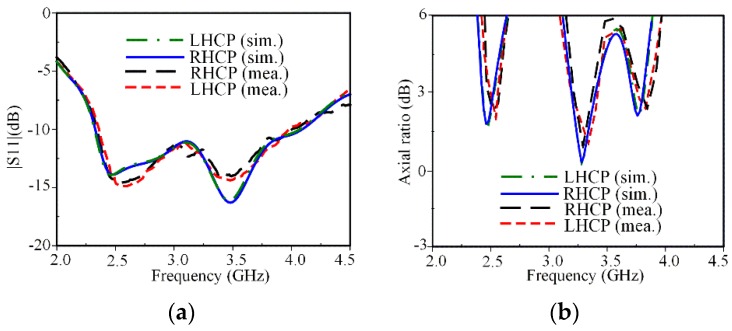
Measured results for both CP states: (**a**) S11; (**b**) axial ratio.

The simulated and measured IBW and ARBW characteristics for both LHCP and RHCP states are shown in Figure 10. The measured IBW for LHCP and RHCP was 52.6% (2.34–4.01 GHz), which agrees well with the simulated result of 55.2% (2.32–4.09 GHz), as shown in Figure 10a. The comparison of the simulated and measured ARBW is shown in Figure 10b. 

**Figure 11:**
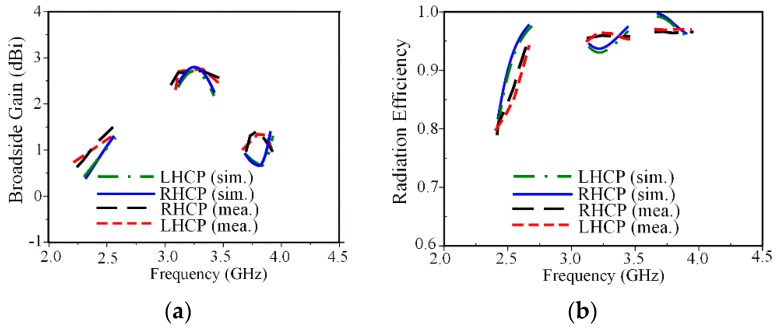
Measured results for both CP modes: (**a**) broadside gain; (**b**) radiation efficiency.

The measured 3-dB ARBWs of 3.5% (2.47–2.56 GHz), 6.6% (3.20–3.42 GHz), and 2.4% (3.74–3.83 GHz) agree well with the simulated results of 4.03% (2.43–2.53 GHz), 6.4% (3.19–3.40 GHz), and 2.4% (3.72–3.81 GHz). Note that the measured ARBWs cover various wireless applications such as: WLAN (2.5 GHz) and some part of the WiMAX medium band (3.2–3.8 GHz). The simulated and measured broadside gain and radiation efficiency within ARBW are shown in Figure 11. The measured broadside gains at frequencies of 2.5, 3.3, and 3.8 GHz are 1.2, 2.7, and 1.4 dB as shown in Figure 11a, respectively. The smaller gains obtained at 2.5 and 3.8 GHz are mainly radiated from the parasitic loop, while the larger gain at 3.3 GHz is caused by radiation from the square-ring radiator. The measured radiation efficiency is over 78% within three CP bands as shown in Figure 11b. 

**Figure 12:**
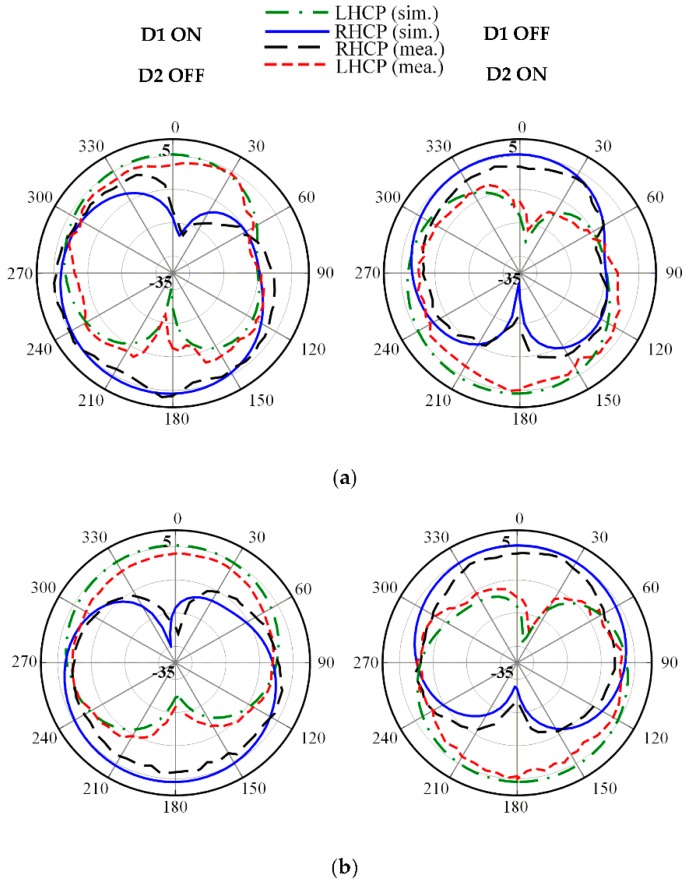
Measured radiation patterns at 2.5 GHz on: (**a**) *x*-*z* plane; (**b**) *y*-*z* plane.

**Figure 13:**
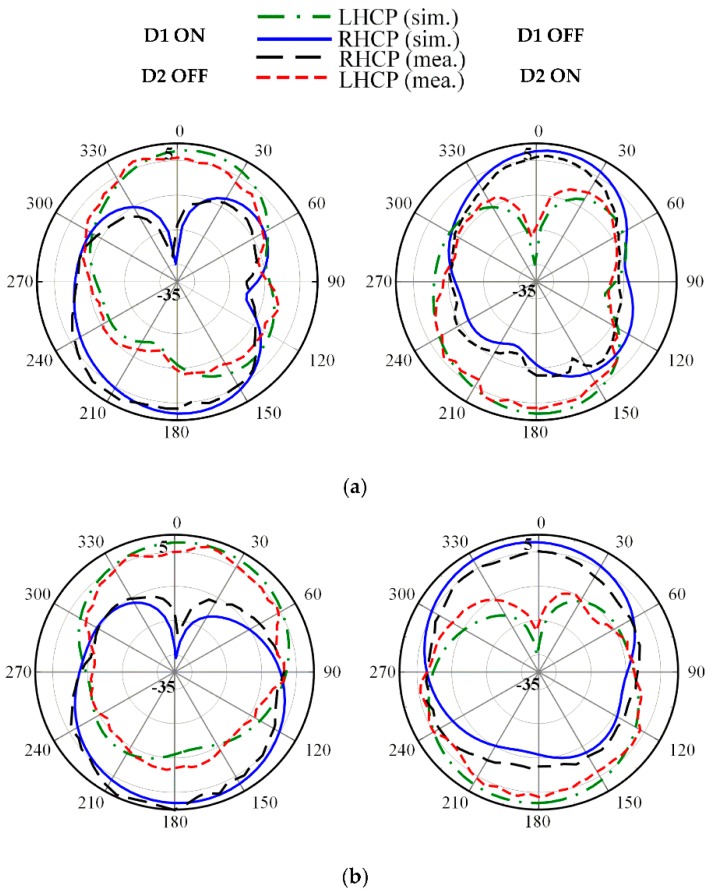
Measured radiation patterns at 3.3 GHz on: (**a**) *x*-*z* plane; (**b**) *y*-*z* plane.

**Figure 14:**
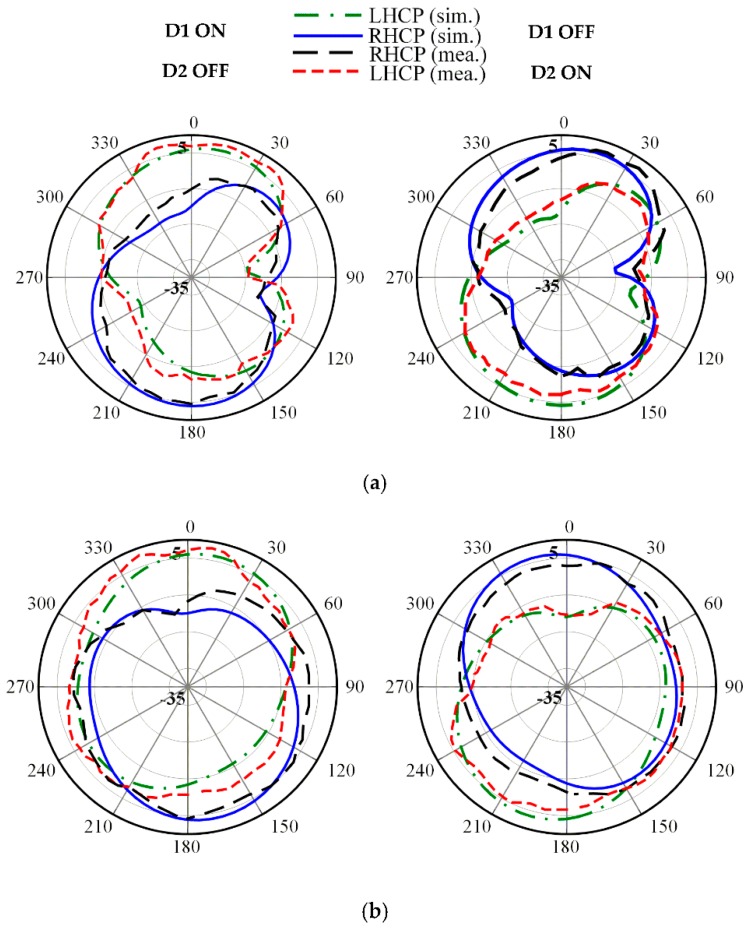
Measured radiation patterns at 3.8 GHz on: (**a**) *x*-*z* plane; (**b**) *y*-*z* plane.

The radiation patterns for both LHCP and RHCP in the principal *x*-*z* and *y*-*z* plane at three frequencies of 2.5, 3.3 and 3.8 GHz are plotted in Figure 12, Figure 13 and Figure 14, respectively. The proposed antenna radiates a bidirectional wave with opposite circular polarization. It can be observed that the proposed antenna is radiating LHCP when *D*_1_ ON and *D*_2_ OFF, and is radiating RHCP when *D*_1_ OFF and *D*_2_ ON in +*z* direction. Overall, reasonable agreement between the simulated and measured results is achieved. 

In addition, the radiation pattern is somewhat titled due to asymmetrical surface current distribution when the DC voltage is applied. Table 3 shows a comparison of the proposed antenna to the other bi-directional multi-band reconfigurable CP antennas. In this table, λ_0_ is the wavelength at the lowest CP frequency point. It can be found that the proposed antenna shows competitiveness in terms of the number of reconfigurable CP bands and diodes. The antenna in [11] has the smallest size but uses four diodes. Meanwhile, the antenna in [12] uses two diodes but the number of reconfigurable CP bands was limited to two. Finally, the proposed antenna obtains reconfigurable CP radiation using only two diodes at three distinct frequencies.

## 4. Conclusions

A simple design for a low-cost, triple-band reconfigurable circularly polarized antenna is discussed. The antenna is able to switch polarization between LHCP and RHCP by DC biasing using two PIN diodes at three different frequencies. The experiment results for both LHCP and RHCP states show that the proposed antenna exhibits a wide IBW of 55.2% (2.32–4.09 GHz). The measured broadside gains are 1.2, 2.7, and 1.4 dB for both LHCP and RHCP states within a measured ARBW of 3.5% (2.47–2.56 GHz), 6.6% (3.20–3.42 GHz) and 2.4% (3.74–3.83 GHz), respectively. The measured radiation efficiency within three CP bands is over 78%. The simulation and measurement results show good agreement. The proposed antenna is suitable for wireless applications such as WLAN (2.5 GHz) and some part of the WiMAX medium band (3.3/3.8 GHz), in which the operating band is tunable by modifying antenna design parameters such as *W*_1_, *W*_2_, and *H*_2_. To the best of the authors’ knowledge, this is the first study to achieve reconfigurable CP radiation at three distinct frequencies using only two diodes.

## Figures and Tables

**Table 1:** CP mode generation from two orthogonal LP modes for each STEP.

	Mode	*f_L_*_1_	*f_U, PL_*	*f_U_*	*f_L_*_2, *PL*_	*f*_*L*2_
STEP						
**STEP 1**	Resonant frequency (GHz)	N/A	N/A	2.7	N/A	N/A
	CP mode (GHz)	N/A				
**STEP 2**	Resonant frequency (GHz)	1.2	N/A	2.8	N/A	3.9
	CP mode (GHz)	2.1* 2.5 3.3				
**STEP 3**	Resonant frequency (GHz)	1.1	2.5	2.8	3.5	4.1
	CP mode (GHz)	1.7* 2.5 3.3 3.8				
**STEP 4**	Resonant frequency (GHz)	1.1	2.5	2.8	3.5	4.0
	CP mode (GHz)	1.7* 2.5 3.3 3.8				

* AR > 3 dB.

**Table 2:** Switching of the CP-sense according to the state of the PIN diodes.

*D*_1_	*D*_2_	2.5 GHz	3.3 GHz	3.8 GHz
ON	OFF	LHCP	LHCP	LHCP
OFF	ON	RHCP	RHCP	RHCP

**Table 3:** Performance comparison with other bi-directional multi-band reconfigurable CP antennas.

Ref.	Size (λ_0_^3^)	Antenna Structure	Diodes/CP Bands
[11]	0.28 × 0.29 × 0.01	Monopole	4/2
[12]	0.31 × 0.31 × 0.01	Monopole	2/2
Proposed	0.56 × 0.44 × 0.01	Monopole	2/3
